# Urothelial carcinoma mimicking Bosniak IV cystic mass: A case report

**DOI:** 10.1016/j.radcr.2025.04.029

**Published:** 2025-04-26

**Authors:** Jinho Jung, Chang Shu, Parvaneh Hassani, Michael Phillipi, Vincent Lee, Roozbeh Houshyar, James Shi

**Affiliations:** a School of Medicine, University of California, Irvine, CA, USA; b University of California, Computational Abdominal Radiology Lab, Orange, CA, USA; c Department of Pathology, University of California, Orange, CA, USA; d Department of Radiology, University of California, Orange, CA, USA

**Keywords:** Case reports, Transitional cell carcinoma, Cysts, Diverticulum, Renal cell carcinoma, Calculi, Cystic kidney diseases, Kidney, Kidney neoplasms

## Abstract

Urothelial carcinoma is the primary malignancy of the urothelium that has varying radiographic features based on the location of the tumor. Differentiating urothelial carcinoma from renal cell carcinoma is critical as interventions and management methods differ. We present a case of urothelial carcinoma within the calyceal diverticula that was initially suspected to represent Bosniak IV cyst due to cystic renal cell carcinoma. A 71-year-old male with a history of gross hematuria and a previously identified Bosniak II renal cyst underwent further imaging, revealing a Bosniak IV cystic mass with enhancing nodules. Subsequent nephrectomy unveiled noninvasive low-grade papillary urothelial carcinoma within a calyceal diverticulum. This case highlights the complexity of diagnosing urothelial carcinoma within the calyceal diverticula, emphasizing the need for a high index of suspicion. The study contributes to understanding the limitations of imaging modalities, especially in cases involving calcification or stone evaluation. The coexistence of urothelial carcinoma and calyceal diverticula is rare but crucial for accurate diagnosis and treatment. Documenting cases like these is vital for recognizing urothelial carcinoma mimics and ensuring appropriate patient management. The study underscores the significance of distinguishing features of calyceal diverticula and advocates for comprehensive imaging approaches in renal cystic lesions.

## Background

Urothelial carcinoma is the primary malignancy of the urothelium. Ninety to ninety-five percent of cases occur in the lower urinary tract (bladder, urethra) while the remaining 5%-10% develop in the upper urinary tract (renal pelvis, ureter). This malignancy most commonly affects the elderly, with a mean diagnosis age of 73 years [1], and predominates in men, who are nearly 4 times more susceptible [2,3]. Environmental factors, notably tobacco smoking, contribute significantly to urothelial carcinoma risk with smokers facing a two to fourfold increased risk when compared to nonsmokers [4]. Prognosis varies markedly and early detection is vital for positive clinical outcomes, with a 5-year survival rate of approximately 70% for localized disease (96% for carcinoma in situ) and a sharp decline to 5% when metastatic [5]. While differentiating urothelial carcinoma from renal cell carcinoma is critical as interventions and management methods differ, current literature emphasizes both the importance and complexity of differentiating an atypical presentation of urothelial carcinoma from Bosniak cysts [6,7].

Here we present a case of urothelial carcinoma within the calyceal diverticula that was initially suspected to represent Bosniak IV cyst due to cystic renal cell carcinoma.

## Case presentation

A 73-year-old male presented to our institution’s urology clinic after outside referral due to complaints of chronic lower back and right mid-flank pain. The patient had a known right renal cyst, initially described as an asymptomatic 2.8 cm parapelvic mass on outside magnetic resonance imaging (MRI) 4 years prior. Four months before referral to our institution, the patient experienced an episode of gross hematuria, prompting further imaging evaluation. Computed tomography (CT) and MRI at that time revealed an increase in the size of the lesion to 4.4 cm. The lesion was described as a renal cyst and classified as Bosniak II with noted calcifications. Subsequent CT imaging 2 months later demonstrated further evolution of the lesion, which was then characterized as a complex Bosniak III cyst measuring 4 cm with an enhancing nodular soft tissue component and layering calcifications.

At the time of presentation to our institution, the patient reported worsening urinary incontinence, increased frequency, and nocturia in addition to flank pain. He denied weak stream or dysuria, and the physical exam was unremarkable. The patient’s past medical history was significant for a traumatic brain injury treated with craniotomy 4 years prior, with the subsequent development of a pulmonary embolism secondary to deep vein thrombosis, necessitating the placement of an inferior vena cava filter. Additional medical history included anemia, benign prostatic hyperplasia, hypertension, and hyperlipidemia. Surgical history was significant for appendectomy. While family history was significant for throat cancer in his father and esophageal cancer in his brother, the patient performed no genetic testing during his medical workup.

**Fig. 1:**
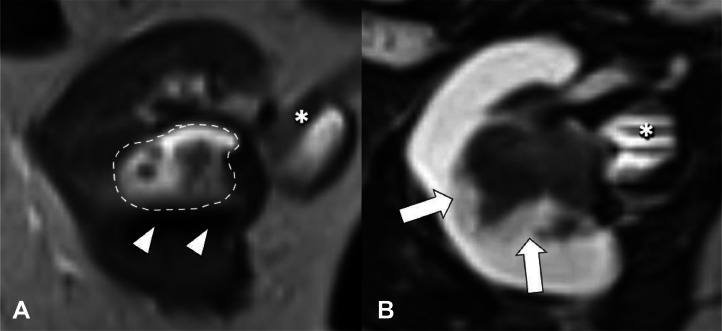
MRI of the right kidney. (A) T2 axial MRI shows hyperintense diverticulum (dotted lines) with hypointense layering calcifications/calculi (white arrowheads) (B) Contrast enhanced axial T1 image show enhancing nodules (white arrows) and contrast excretion into the renal pelvis (white *).

While the patient’s gross hematuria had resolved since the prior episode, microscopic hematuria persisted. Laboratory evaluation was unremarkable; the patient’s blood urea nitrogen, creatinine, and estimated glomerular filtration rate were within references ranges. Repeat MRI revealed increase in size of the lesion to a 4.3 cm Bosniak IV cyst located in the mid to interpolar cortical region of the right kidney with multiple peripheral enhancing nodules (Fig. 1). There was no evidence of metastatic disease. Urine cytological analysis was negative for high-grade urothelial carcinoma but demonstrated numerous red blood cells.

**Fig. 2:**
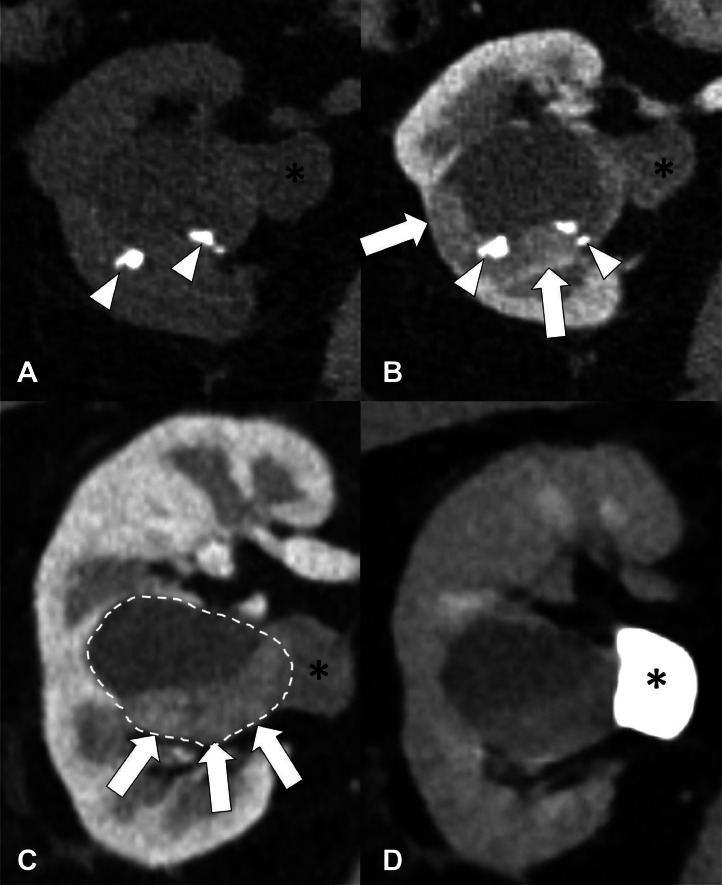
CT of the right kidney. (A) Noncontrast axial CT shows a cystic lesion with a few layering calcifications/calculi (white arrowheads). (B and C) contrast enhanced axial and coronal images show a peripheral rim of enhancing soft tissue material (white arrows) in the cystic lesion (dotted lines). (D) Delayed excretory phase coronal post contrast CT shows contrast excretion into the renal pelvis (black *) and not within the cystic lesion (dotted line).

**Fig. 3:**
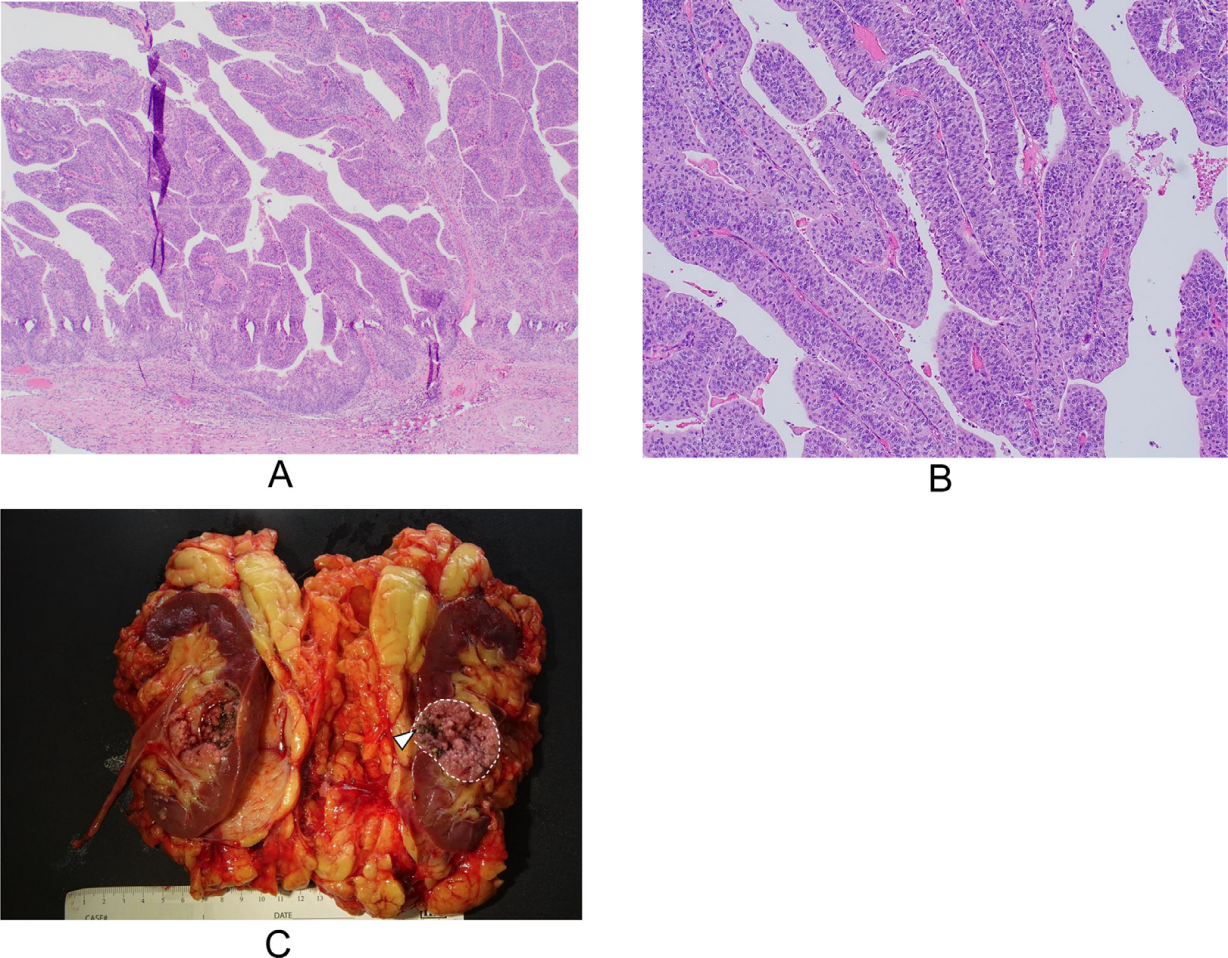
(A) Histologic examination shows fused to branching fibrovascular cores lines by neoplastic urothelium. From low power, architecture is relatively organized and cytology appears uniform, supporting a low-grade lesion. There is no invasion past the basement membrane. (B) From high power, there is slight variation in size and shape of nuclei, but in general polarity is maintained. Mitoses are rare. These features support the diagnosis of low-grade papillary urothelial carcinoma. (C) Macroscopic surgical specimen of urothelial carcinoma (dotted lines) with layering calcifications (white arrowhead).

In a review of the case by our institution’s Tumor Board, the lesion was characterized as a hilar mass in the interpolar region with progressive enlargement. The MRI findings were deemed consistent with the appearance of cystic renal cell carcinoma. Based on these findings, the Tumor Board recommended a robotic right nephrectomy for definitive management. A follow up preoperative CT urogram demonstrated a stable Bosniak IV cystic mass but did also note posterior calcifications within the lesion (Fig. 2). However, pathologic examination of the renal mass after nephrectomy yielded an unexpected diagnosis of noninvasive low-grade papillary urothelial carcinoma within a calyceal diverticulum (Fig. 3). Given the discrepancy between the radiological and pathological findings, the Tumor Board reconvened to review the case and determine the appropriate management strategy. The Board ultimately recommended either observation, endoscopic management, or ureterectomy. The patient opted for observation, which consisted of a 6-month follow-up CT urography. At the time of the first follow up, the patient denied any further urinary symptoms, including hematuria, flank pain, or back pain. CT urography demonstrated expected postsurgical changes of right nephrectomy without suspicious findings at the nephrectomy bed or evidence of metastatic disease. Concurrent urine cytology was negative for high-grade urothelial carcinoma. The patient is currently scheduled for continued urography and cytology follow-up every 6 months.

## Discussion

This unique case details how a speciously described Bosniak IV cystic mass was discovered to be urothelial carcinoma arising within a calyceal diverticulum. The lesion was initially classified using the Bosniak criteria due to the assumption that the fluid component was a cystic mass. What was thought to be calcifications within the cystic mass were small layering stones within the calyceal diverticulum. In this context then, the enhancing nodular component was the primary urothelial neoplasm as opposed to what was believed to be a cystic renal cell carcinoma. In retrospect, the tumor obstructed the entrance into the diverticulum, which is why it did not fill with contrast on delayed imaging. This study builds on previous reports which underscores both the importance and complexity of differentiating an atypical presentation of urothelial carcinoma from Bosniak cysts [6,7].

Radiographic features of urothelial carcinoma vary based on the location of the tumor. For example, computed tomography (CT) of urothelial carcinoma within the ureter or urethra typically demonstrates focal soft tissue thickening. Urothelial carcinoma of the renal pelvis begins in the pelvis and can subsequently invades the renal sinus fat or renal parenchyma [8,9]. Compared to conventional clear cell renal cell carcinoma, urothelial carcinoma is hypoenhanceing [10,11]. Urothelial carcinoma also maintains the renal contour regardless of infiltration severity unlike renal cell carcinoma, which is typically exophytic and dramatically alters the renal contour. Differentiating urothelial carcinoma from renal cell carcinoma is critical as interventions and management methods differ. Specifically, patients with renal cell carcinoma undergo partial to total nephrectomy, whereas patients with urothelial carcinoma undergo total nephroureterectomy [12].

Calyceal diverticula are rare outpouchings of the renal pelvis or calyx into the renal parenchyma. They fall into 2 categories: type I, which connects with a minor calyx or an infundibulum, and type II, which originates from the renal pelvis or a major calyx. Type II diverticula, situated in the central region of the kidney, are larger, more likely to exhibit symptoms, and tend to be more prominent [13].

For a majority of cases, patients are asymptomatic, and the findings are discovered incidentally through imaging [14]. If symptomatic, patients can present with hematuria, calculi, flank pain, and/or recurring infection. On noncontrast CT, calyceal diverticula appear as fluid density structures mimicking cysts. A calyceal diverticulum can be best distinguished from a simple renal cyst through delayed contrast imaging: calyceal diverticula should fill with contrast while a renal cyst, unconnected from the collecting system, does not fill with contrast. The coexistence of a calyceal diverticulum and urothelial carcinoma represents a rare clinical scenario, thus recognition and appropriate intervention requires a high index of suspicion. While the incidence of calyceal diverticula is low, the frequency of stone formation within them is high. In 9.5% to 50% of reported cases, stones have been discovered. When considering combined series, this figure increases to 96%, with an average stone size of 12.1 mm, ranging from 1 to 30 mm [15]. In this case, the small, round, uniform stones within the cystic structure could have suggested the possibility of a calyceal diverticulum despite the lack of contrast opacification due to obstruction by the tumor.

Calyceal diverticula typically contain several small calculi rather than a single large one. When the patient is in a supine position, multiple small stones within a diverticulum tend to layer along its posterior wall. If a sonographer is not familiar with this condition, a calyceal diverticulum might be mistakenly interpreted as a complex cystic lesion on ultrasound. Therefore, it is recommended that when echogenic material or posterior wall calcification is observed within a renal cystic lesion, the patient should be scanned in both the supine and prone positions to determine whether there are mobile calculi present within a calyceal diverticulum [16].

The Bosniak classification system is widely used to characterize and stratify cystic renal lesions into risk categories based on imaging features [15,16]. In 2019, Silverman et al proposed an update that classifies renal cysts based not only on CT but also magnetic resonance imaging (MRI) [17]. Bosniak I and II categories include benign simple and minimally complex cysts. Bosniak IIF lesions demonstrate increased complexity, with either minimally thickened wall or septation or many (≥4) thin septations [15,16,18]. Bosniak III lesions are indeterminate and require further evaluation due to their intermediate risk for malignancy. They exhibit enhancing thick of irregular walls and/or septations. Lastly, Bosniak IV lesions are highly suspicious for malignancy, characterized by enhancing nodule(s).

## Conclusion

This case demonstrates urothelial carcinoma within a calyceal diverticulum mimicking a cystic renal mass. It is important to be familiar with the distinguishing features of diverticula, in particular the unique appearance of the stones that form within the structure. This case also highlights one of the potential limitations of MRI for characterizing cystic renal masses, which is calcification or stone evaluation. Documentation of this case may elucidate other instances of urothelial carcinoma mimics, leading to appropriate diagnosis and treatment of these patients.

## Ethics approval and consent to participate

No ethical approval was required as this manuscript is a case report.

## Consent for publication

Written informed consent was obtained from the patient for publication of this case report and any accompanying images. A copy of the written consent is available for review by the Editor-in-Chief of this journal.

## Availability of supporting data

The dataset supporting the conclusions of this article is available upon request to the corresponding author.

## Author contributions

All authors have all contributed as authors to this manuscript in terms of planning, conception, and design, writing and editing various drafts of the manuscript and read and approved the final manuscript.

## Patient consent

A written and informed consent was obtained from the patient for publication of this case report.
